# Evaluating the effect of chromosomal context on zinc finger nuclease efficiency

**DOI:** 10.1186/1753-6561-7-S6-P3

**Published:** 2013-12-04

**Authors:** Scott Bahr, Laura Cortner, Sara Ladley, Trissa Borgschulte

**Affiliations:** 1 CHOZN® Platform Development Team, SAFC/Sigma-Aldrich, St Louis, MO 63103, USA

## Introduction

Zinc Finger Nuclease (ZFN) technology has provided researchers with a tool for integrating exogenous sequences into most cell lines or genomes in a precise manner. Using current methods, the efficiency of targeted integration (TI) into the host genome is generally low and is highly dependent on the ZFN activity at the genomic locus of interest. It is unknown if the ZFN binding and cutting efficiency is more dependent on the nucleotide recognition sequence or the chromosomal context in which the sequence is located.

We have taken a highly efficient ZFN pair (hAAVS1) from human studies and introduced the exogenous DNA sequence into the Chinese Hamster Ovary (CHO) genome in an attempt to improve the efficiency of targeted integration. A "Landing Pad" comprised of human AAVS1 sequence has been integrated into the CHO genome at 3 separate loci to determine if the ZFN's will work across species and if the cutting efficiency is affected by chromosomal context. The results of this study will help us to improve the overall efficiency of TI by using Landing Pads, particularly for genomic targets in which suitable ZFN's may not be available.

## Methods

**Figure 1:**
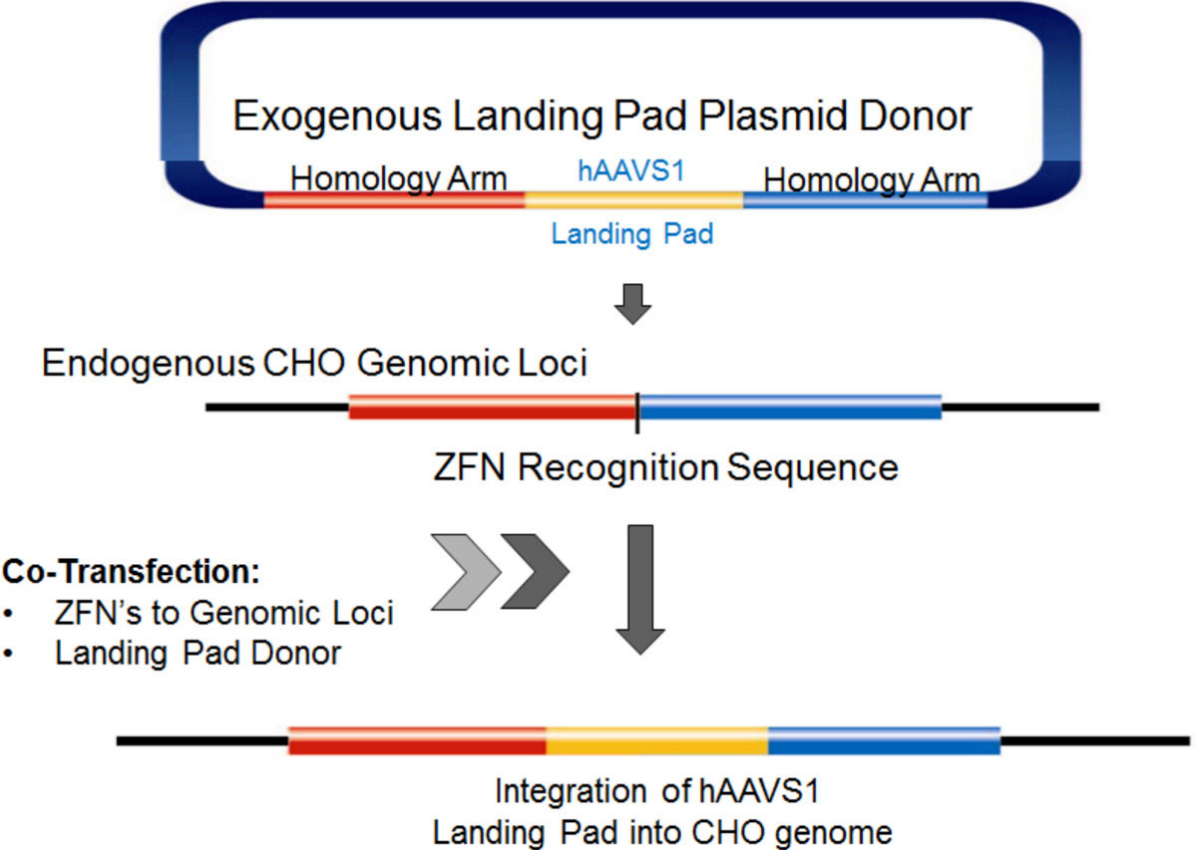
**Schematic of ZFN mediated Integration of the hAAVS1 Landing Pad into CHO**.

3 CHO Loci were chosen for this study based on previous gene expression studies. Rosa26 and Neu3 show consistent but low levels of expression while Site #1 appears to have no known coding sequence. Additionally, Rosa26 and Site#1 were chosen as potential safe harbor sites in CHO. The ZFN cutting efficiency at the endogenous CHO loci Rosa26, Site #1 and Neu3 are approximately 15%, 30% and 40% respectively. Based on other studies the cutting efficiency of human AAVS1 ZFN's was as high as 50% depending on the human cell line used. A plasmid donor carrying the hAAVS1 ZFN recognition sequence Landing Pad was introduced into CHO Rosa26, Site #1, and Neu3 via targeted integration (Figure 1).

## Results

Clones carrying the exogenous hAAVS1 Landing Pads at Rosa26, Site #1 and Neu3 were transfected with hAAVS1 ZFN's and the cutting efficiency was measured. We found that the human AAVS1 ZFN's were able to successfully cut at their recognition sequence in the Landing Pad at all 3 CHO loci to varying degrees (Table 1). ZFN efficiency at each loci was measured by Cel1 Assay or direct sequencing of Indels in PCR amplicons. We see successful ZFN activity at all 3 loci but with varying efficiency. **The Landing Pad integration at Neu3 locus caused phenotypic changes in the cell growth and viability following transfection which may explain low ZFN activity.

**Table 1:** Comparing ZFN activity in CHO before and after Landing Pad Integration

CHO Site	ZFN Activity at Endogenous CHO Locus	ZFN Activity at Integrated Landing Pad
**Rosa 26**	16%	18%

**Site #1**	31%	51%

**Neu3**	41%	16%**

## Conclusions

These results indicate that the chromosomal context of the ZFN recognition sequence has an effect on cutting efficiency. This study shows that TI can be performed with Landing Pads across species with high efficiency and provide researchers with additional tools for cell line engineering. Further development of Landing Pads could create highly engineered and multi-functional platforms that would facilitate more efficient and more tailored CHO cell modifications.

